# Delineating Chromosomal Breakpoints in Radiation-Induced Papillary Thyroid Cancer

**DOI:** 10.3390/genes2030397

**Published:** 2011-06-28

**Authors:** Heinz-Ulrich G. Weier, Yuko Ito, Johnson Kwan, Jan Smida, Jingly F. Weier, Ludwig Hieber, Chun-Mei Lu, Lars Lehmann, Mei Wang, Haig J. Kassabian, Hui Zeng, Benjamin O'Brien

**Affiliations:** 1 Life Sciences Division, E.O. Lawrence Berkeley National Laboratory, 1 Cyclotron Road, Berkeley, CA 94720, USA; E-Mails: ugweier@lbl.gov (H.-U.G.W.); kwanj@mail.amc.edu (J.K.); hjkassabian@gmail.com (H.J.K.); hzeng@lbl.gov (H.Z.); 2 National Institute of Science and Technology Policy (NISTEP), Ministry of Education, Culture, Sports, Science and Technology, Tokyo 100-0005, Japan; E-Mail: itoh@nistep.go.jp; 3 Clinical Cooperation Group Osteosarcoma, Helmholtz Zentrum München, German Research Center for Environmental Health, Ingolstädter Landstrasse 1, 85764 Neuherberg, Germany; E-Mail: smida@helmholtz-muenchen.de; 4 Clinical Labs–Cytogenetics, University of California, 185 Berry Street Suite 290, San Francisco, CA 94143-0100, USA; E-Mail: jinglyw@gmail.com; 5 Department of Radiation Cytogenetics, Helmholtz Zentrum München, German Research Center for Environmental Health, Ingolstädter Landstr.1, Neuherberg 85764, Germany; E-Mail: ludwig.hieber@helmholtz-muenchen.de; 6 Department of Chemical and Materials Engineering, National Chin-Yi University of Technology, No.35, Lane 215, Section 1, Chungshan Road, Taiping City, Taichung 411, Taiwan; E-Mail: lucm@ncut.edu.tw; 7 Roche Diagnostics GmbH, Nonnenwald 2, 82377 Penzberg, Germany; E-Mail: lars.lehmann@roche.com; 8 Department of Diabetes, City of Hope, 1500 Duarte Road, Duarte, CA 91010-3012, USA; E-mail: mwang@coh.org; 9 William Harvey Research Institute, Translational Medicine and Therapeutics, Barts and The London School of Medicine, Charterhouse Square, London, EC1M 6BQ, UK; 10 Department of Anesthesiology, German Heart Institute Berlin, Augustenburger Platz 1, 13353 Berlin, Germany

**Keywords:** Chernobyl, neoplastic disease, papillary thyroid cancer, translocation, molecular cytogenetics, breakpoint delineation, fluorescence *in situ* hybridization, bacterial artificial chromosomes

## Abstract

Recurrent translocations are well known hallmarks of many human solid tumors and hematological disorders, where patient- and breakpoint-specific information may facilitate prognostication and individualized therapy. In thyroid carcinomas, the proto-oncogenes RET and NTRK1 are often found to be activated through chromosomal rearrangements. However, many sporadic tumors and papillary thyroid carcinomas (PTCs) arising in patients with a history of exposure to elevated levels of ionizing irradiation do not carry these known abnormalities. We developed a rapid scheme to screen tumor cell metaphase spreads and identify candidate genes of tumorigenesis and neoplastic progression for subsequent functional studies. Using a series of overnight fluorescence *in situ* hybridization (FISH) experiments with pools comprised of bacterial artificial chromosome (BAC) clones, it now becomes possible to rapidly refine breakpoint maps and, within one week, progress from the low resolution Spectral Karyotyping (SKY) maps or Giemsa-banding (G-banding) karyotypes to fully integrated, high resolution physical maps including a list of candiate genes in the critical regions.

## 1. Introduction

It is becoming increasingly clear that the pathogenesis of radiation-induced tumors is often distinctly different from that of spontaneous, non-radiation-induced tumors. Our research focuses on the physical mapping of proto-oncogenes related to tumorigenesis such as the neurothrophic growth factor receptor 1, NTRK1 (also known as trk-A) [1], the development of assays to detect chromosomal rearrangements leading to activation of oncogenes [2–5], and the mapping of translocation breakpoints in spontaneous cases of PTC as well as tumors in patients with a known history of either therapeutic or accidental exposure to ionizing radiation [6–8].

While chromosomal rearrangements activating NTRK1 are relatively rare and not a marker of exposure to ionizing radiation [9–12], the situation is different in cases with mutations involving the cadherin-family associated cell surface receptor, RET, another receptor-type tyrosine kinase (rtk) gene, found on Chromosome 10 [2–5,13–16].

Numerous studies could demonstrate a correlation between exposures to ionizing radiation and particular RET/PTC rearrangements *in vivo* leading to the expression of chimaeric proteins [17–22].

Fluorescence *in situ* hybridization is one of the most powerful tools to detect these genetic aberrations underlying the expression of chimaeric proteins [23–25]. Such proteins alter the signaling pathways in cells that have undergone neoplastic transformation [26–28].

Table 1 gives an overview of the most relevant RET/PTC rearrangements analyzed and described to date.

**Table 1:** RET/papillary thyroid carcinoma (PTC) rearrangements.

***RET type***	***Partner Gene***	***Chromosomal positions***	***Reference***
RET/PTC 1	H4 (CCDC6, D10S170)	inv10(q11.2;q21)	[29]
RET/PTC 2	PRKAR1A	t(10;17)(q11.2;q23)	[30]
RET/PTC3; RET/PTC4	NCOA4	inv10(q11.2;q10)	[18,31]
RET/PTC5	GOLGA5 (RFG5)	t(10;14)(q11.2;q32)	[32]
RET/PTC6	TRIM24 (HTIF1)	t(7;10)(q32–34;q11.2)	[20]
RET/PTC7	TIF1G (RFG7,TRIM33)	t(1;10)(p13;q11.2)	[20]
ELKS-RET	ELKS (RAB6IP2)	t(10;12)(q11.2;p13.3)	[33]
RET/PTC8	KTN1	t(10;14)(q11.2;q22.1)	[34]
RET/RFG9	RFG9	t(10;18)(q11.2;q21–22)	[35]
PCM1-RET	PCM1	t(8;10)(p21-22;q11.2)	[36]
RFP-RET	RFP (TRIM27)	t(6;10)(p21;q11.2)	[37]
HOOK3-RET	HOOK3	t(8;10)(p11.21;q11.2)	[38]

More recent studies have shown that the most common RET/PTC1 and RET/PTC3 rearrangements map to the known fragile site FRA10G [39] and can be created *in vitro* with fragile site-inducing chemicals such as aphidicolin [40].

**Figure 1:**
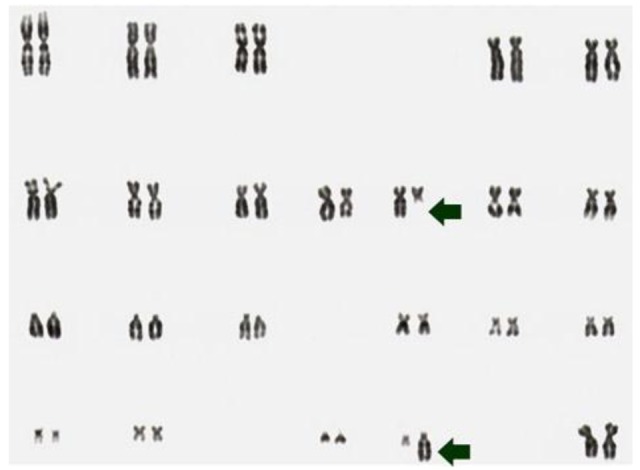
G-banding karyotype of the PTC cell line S96T. The arrows point at the chromosomes involved in the apparently balance reciprocal translocation t(10;22)(q11;q11).

Despite a high prevalence of mutations or rearrangements activating the rtks NTRK1 or RET, many phenotypically similar tumors do not show this abnormality. Adding a further level of complexity, in our studies of post-Chernobyl cases of PTC, only few tumors showed clonal abnormalities in 100% of metaphase spreads like the case S96T (Figure 1) [7,8,25].

We hypothesize that these normal-looking metaphase spreads carry small submicroscopic lesions also known as cryptic translocations that are missed by the conventional methods of metaphase cell analysis, *i.e.*, G-banding, whole chromosome painting (WCP) or SKY [8,41–43].

Therefore, we feel that it is necessary to combine a variety of cytogenetic techniques to comprehensively describe all relevant aberrations. To further explore this, we utilized cell lines established from three cases of radiation-induced childhood thyroid cancer: S96T, as mentioned above, and S47T and S48T, as analyzed further in this publication.

Table 2 gives an overview over clinical details and findings from G-banding and FISH studies in these cell lines.

**Table 2:** Clinical details and findings from G-banding and FISH studies in the three cases discussed in this communication.

**Case**	**Gender**	**Age at Surgery**	**Age at Accident**	**G-Banded Metaphase Cells**	**Result**	**SKY Results**	**Activated tk Gene**	**Reference**
S47T	female	13 years	6 years	10	t(5;7)(q23;p15)	t(5;7)	RET/PTC3	[17]
S48T	male	14 years	7 years	6	Multiple (see text)	der(1;4),der(1;6),der(1;6;11),der(2;17),der(2;6;11),der(2;7;11),der(2;11;17),der(3;8),der(3;9),der(6;11),der(7;9;15),der(9;13)	NTRK1	[8,44]
S96T	female	14 years	6 years	10	t(10;22)(q11;q11)	Not determined	--	[8]

The RET/NTRK1 status of the cell lines used in this study has been published previously [17,34,45].

Now, if these oncogenic events arise from balanced intra- or interchromosomal rearrangements, gene copy numbers remain unchanged compared to normal diploid cells, and comparative genomic hybridization assays using either metaphase spreads [46], oligonucleotide (Nimblegen; Affymetrix) or bacterial artificial chromosome arrays [47,48] will fail to detect the abnormalities.

**Figure 2:**
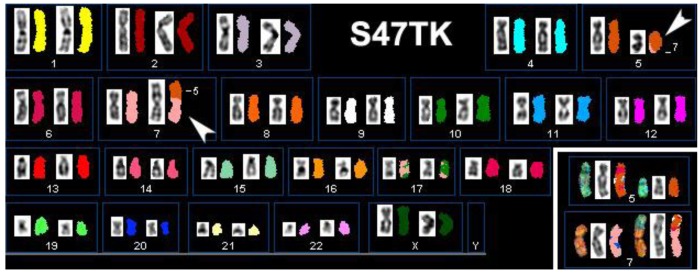
Spectral Karyotype analysis of the PTC cell line S47T. The arrowheads point at the abnormal chromosomes derived from the t(5;7)(q23;p15). The insert shows derivative chromosomes from a metaphase spread that did not show chromosome 7 material on the der(5).

An additional complication in the definition of candidate genes for thyroid tumorigenesis is the great variety in levels of heterogeneity found in primary cell cultures and even established cell lines. Figure 2 illustrates this by presenting the results of our SKY analysis of case S47T, a childhood case of post-Chernobyl PTC [8]. Roughly half of the S47T metaphase spreads that we analyzed by SKY showed a balanced, reciprocal translocation t(5;7)(q23;p15). The other spreads did not show chromosome 7 material translocated to the der(5) (Figure 2, insert).

Similar challenges have been identified in previous publications analyzing PTC-associated rearrangements with and without exposure to ionizing radiation.

Thus, we have to accept that no single cytogenetic technique will reliably detect all potential aberrations found in the pathogenesis of radiation-induced (or indeed spontaneous) tumors.

In this communication we propose an algorithm utilizing a combination of cytogenetic techniques of increasing resolution to comprehensively, expeditiously and cost-effectively delineate chromosomal breakpoints in radiation-induced papillary thyroid carcinomas. By utilizing publicly available resources, our aim was the development of a replicable, targeted approach to breakpoint analysis which can be used by non-specialist laboratories worldwide.

## 2. Results and Discussion

Where significant heterogeneity is observed in cultured cell lines, such as in the case of S47T, the possibility of contamination has to be considered. However, we exclude the possibility of a contamination of these 2 cell lines (S47T and S96T) based on the fact that all 10 out of 10 G-banded metaphases showed the identical translocation (Table 2). Therefore, the fact that individual metaphase spreads prepared from S47T showed two different der(7) chromosomes in subsequent passages of S47T must be due to a deletion event that followed the reciprocal t(5;7) translocation.

Instead of immunofluorescence characterization of cell lines, we performed comprehensive cDNA hybridization experiments. This elucidated DNA changes not visible by SKY or G-banding techniques. Results from these studies have been published [7,17,45].

To develop and validate our algorithm, we focused our attention on cell line S48T.

**Figure 3:**
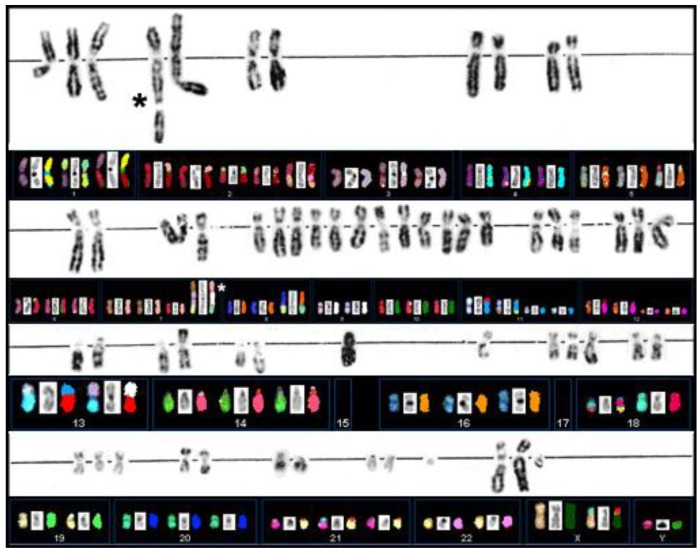
G-banding and SKY analysis of PTC cell line S48T. Spectral Karyotype analysis of PTC line S48T. The asterisks point at the abnormal chromosomes derived from the t(7;9;15).

Extensive G-banding analysis performed in the laboratories in Munich had indicated that primary cultures derived from case S48T carried multiple chromosomal abnormalities. The rearrangements were large in number and mostly unbalanced, which greatly complicated conventional karyotyping based on G-banding analysis (Figure 3) [49]. The cloning of cell line S48T has been described previously [42].

Our Spectral Karyotyping analysis (SKY), shown in Figure 3 below the G-banding results, provided some additional clues to the origin of marker chromosomes.

Cell line S48T did not display signs of rearranged chromosomes 10, but a number of marker chromosomes carrying material from either chromosome 1 or 9 caught our attention. The long arm of chromosome 1 harbors the neurotrophic growth factor receptor kinase-1 (NTRK1) gene [1], which has been reported to be aberrantly expressed in various solid tumors among them post-Chernobyl PTC [9,50].

**Figure 4:**
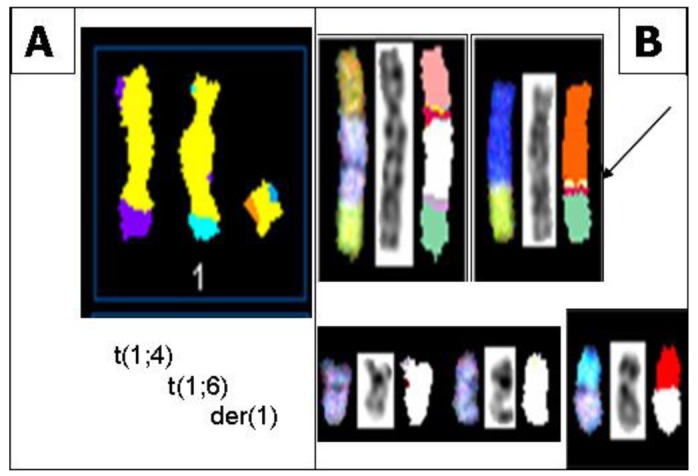
(**A**) SKY classification images of abnormal metaphase chromosomes from S48T containing genetic material derived from chromosome 1. The images show from left to right a t(1;4), a t(1;6) and a der(1) chromosome; (**B**) SKY classification images of abnormal metaphase chromosomes from S48T containing genetic material derived from chromosome 9. The arrow points at the small insertion of chromosome 9 material into a der(8)t(8;15) chromosome that we analyzed in more detail [42].

In all S48T metaphase spreads, we found several marker chromosomes containing genetic material from either chromosome 1 or 9. These common markers, three of which are derived from chromosome 1 (Figure 4A) and four types derived from chromosome 9 (Figure 4B), are shown in Figure 4.

**Figure 5:**
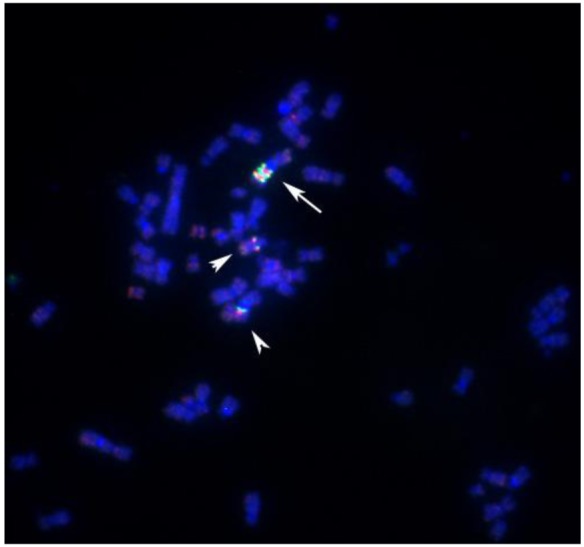
BAC-FISH analysis of the distribution of chromosome 1-derived material in metaphase spreads from cell line S48T. The arrow points at a larger chromosome that carries an amplified region derived from the proximal long arm of chromosome 1. The arrowheads point at the two other der(1) chromosomes.

Protein tyrosine kinases have been implicated in tumor initiation and progression [51–53]. In gene expression studies reported elsewhere, we were able to demonstrate that cell line S48T expresses the tyrosine kinase domain of NTRK-1 [44], which is normally located on the long arm of chromosome 1, band q12-21 [1] at position 156,830,671–156,851,642 bp in the UC Santa Cruz (UCSC) genome browser. For the analysis of chromosome 1 rearrangements, we pooled three individual BAC probes, since this has resulted in more reliable FISH signals [45,54,55]. Hybridization of a combination of a biotinylated probe DNA pool that maps close to NTRK1 at chromosome 1q12-21 (clones RP11-37N10, RP11-71P2 and RP11-315I20) and a digoxigenin–labeled probe pool comprised of probes RP11-262A11, RP11-299D6 and RP11-243J18 that bind close to non-muscle tropomyosin 3 (TMP3) (UCSC position 1: 154,127,780–154,155,725), a known translocation partner of NTRK1 in solid tumor cell lines [50,56], revealed complex translocation and genome amplification in line S48T (Figure 5). Two derivative chromosomes each carried 1 copy of the ∼10 Mbp region flanked by our probe pools (arrowheads in Figure 5), while a large marker chromosome contained about 2.5 copies (arrow in Figure 5).

The results shown in Figure 5 confirm comparative genomic hybridization results that indicated genomic amplification of the proximal long arms of chromosome 1 and chromosome 9 in S48T [42].

**Figure 6:**
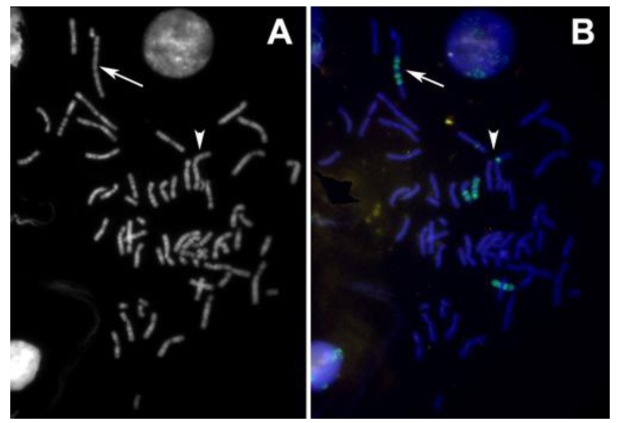
(**A**) The DAPI image of an interphase and a spread metaphase cell from cell line S48T; (**B**) Hybridization of a whole chromosome painting probe specific for chromosome 9 highlights the chromosomes that carry chromosome 9-derived material. The arrows in Figure 6 (**A**) and (**B**) point at the large t(7;9;15) marker chromosome; arrowheads point at the small insertion that we analyzed.

**Figure 7:**
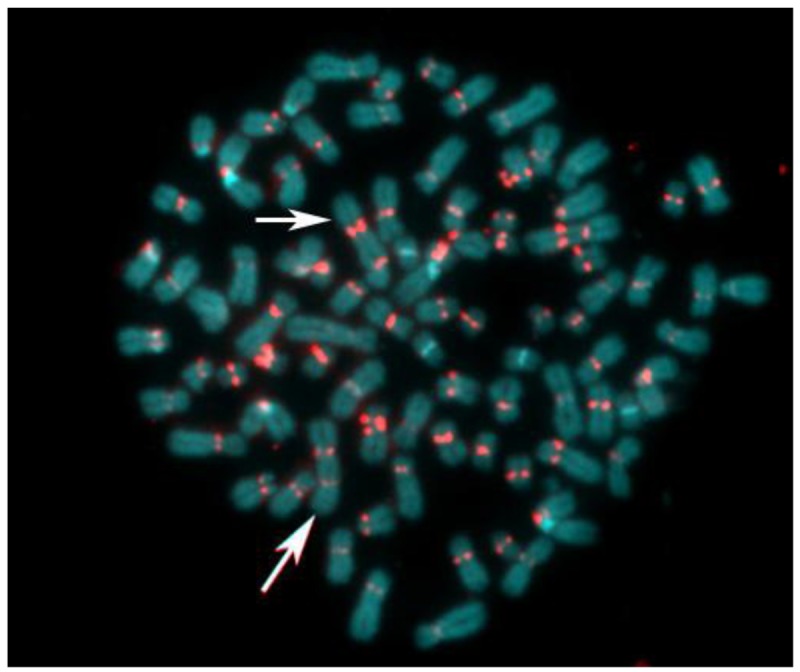
Pan-centromeric staining via *in situ* hybridization using an alpha satellite DNA con-sensus sequence probe reveals the presence of large dicentric chromosomes in this metaphase spread from cell line S48T. The chromosomes were counterstained with DAPI.

The abnormal staining pattern of the large marker chromosome (arrows in Figure 6 A,B) prompted us to investigate the distribution of centromeric heterochromatin in this cell line. Considered a rather rare event, some of the S48T metaphase spreads hybridized with the alpha satellite DNA probe showed not just one, but two large dicentric chromosomes (Figure 7, arrows).

**Figure 8:**
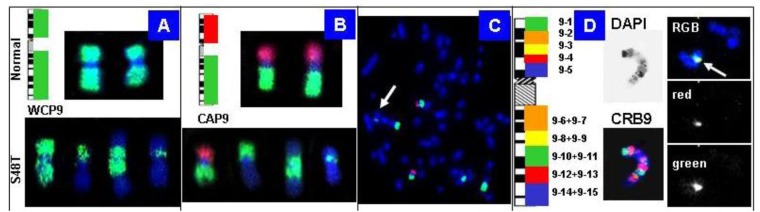
Chromosome 9-specific BAC pools for BAC-FISH. (**A**) Labeling of all clones with the same reporter molecule creates a whole chromosome painting (WCP) probe; (**B–C**) Chromosome arm probes (CAP) provide first clues to the origin of markers. The arrow in the S48T metaphase in (**C**) points to the small insertion; (**D**) Chromosomal rainbow probes for chromosome 9 (CRB9) allowed us to narrow down the origin of the inserted material to chromosome 9, pools 10–11 (right) [42].

Our strategy to rapidly map chromosomal breakpoints in metaphase spreads is based on hybridization of increasingly smaller BAC-derived DNA probe pools. Figure 8 shows chromosome 9-specific examples: the top in (Figure 8A, B) shows the results obtained with normal metaphase chromosomes, whereas the bottom shows chromosomes in S48T.

It should be noted that BAC-FISH is a very sensitive approach to detect translocations [57].

**Figure 9:**
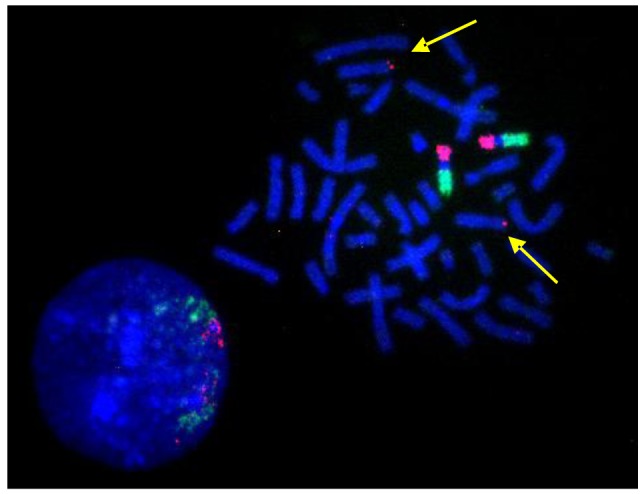
BAC-FISH results suggest a detection-sensitivity in the order of single BAC clones or translocated genomic regions in the order of a few hundred kb. The yellow arrows point at the signal generated by a chromosome 8ptel-specific BAC clone that was cohybridized with the chromosome 9 specific BAC CAP probe sets.

A single BAC clone is sufficient to highlight a small translocation as shown in the example in Figure 9. Here, the BAC clone set contained one sub-telomeric clone that had been assigned by mistake to chromosome 9ptel in one of the databases. As the hybridization experiments showed, this clone maps to the telomere on the short arm of chromosome 8 instead (Figure 9).

**Figure 10:**
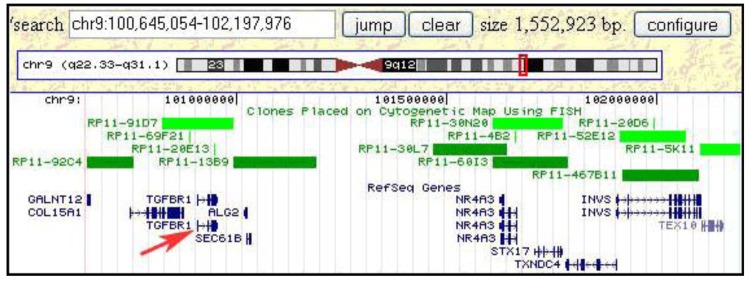
Integrating FISH mapping results and genomic databases rapidly leads to the definition of candidate tumor genes. The figure shows a genomic region of about 1.5 Mbp that was found inserted into a t(8;15) chromosome.

Once a minimal breakpoint interval defined by a single BAC clone or a contig of 2–3 clones is defined, genome databases can be consulted to search for candidate tumor-related genes. For the small insertion into the t(8;9;15) chromosome in S48T this approach is illustrated in Figure 10. This screen dump from the Genome browser web page at the University of California, Santa Cruz (UCSC), shows a region of roughly 1.5 Mbp, which was found inserted into the marker chromosome. Clones that were used in our hybridization experiments are included in the set of FISH mapped clones shown in this Figure (*i.e.*, RP11-92C4, RP11-91D7)

Interestingly, this region from the long arm of chromosome 9 contains the tumor growth factor (TGF) beta receptor 1 (TGFBR1) gene, which when mutated or duplicated, alters the transmission of the subcellular TGF beta signal and has been reported to cause a dominant disease phenotype [48,58]. While these findings do not support a notion that TGF beta duplications have a causal relationship to post-Chernobyl PTC, the observed gain might very well alter the cells' phenotype increasing their chances of survival and increased proliferation in the tumor microenvironment. Conversely, this metabolomic change might become a tumor's Achilles heel in efforts to devise more efficient anti-tumor therapies.

## 3. Experimental Section

### 3.1. Cell Cultures and Preparation of Metaphase Spreads

Normal human control metaphase spreads were made from phytohemagglutinin-stimulated short-term lymphocyte cultures of blood obtained from a healthy male according to the procedure described by Harper and Saunders [59]. Acetic acid-methanol fixed lymphocytes were dropped on ethanol-cleaned slides in a CDS-5 Cytogenetic Drying Chamber (Thermatron Industries, Inc, Holland, MI) at 25 °C and 45–50% relative humidity.

The PTC cultures were established as described by Lehmann *et al.* and Zitzelsberger *et al.* [7,8]. All procedures followed protocols approved by the LBNL/UC Berkeley Institutional Review Board (IRB) Committee on protection of Human Subjects in Research regarding use of surplus surgical tissues for research. S48T lines were obtained from the tumor tissue of a 14 year old patient (7 years at time of exposure to elevated levels of radiation) undergoing surgery at the Center for Thyroid Tumors in Minsk, Belarus, following the diagnosis of Hashimoto's thyroiditis and PTC. Initial chromosome preparations were carried out after an *in vitro* culture of S48T cells for 8–21 days. Later, clones were isolated by limiting dilution and cultured for more than 20 passages. After G-banding with Wright's staining solution, karyotypes were recorded according to the International System for Human Cytogenetic Nomenclature [60].

### 3.2. Comparative Genomic Hybridization (CGH)

Comparative genomic hybridization [46] with DNA isolated from the primary culture as well as cell lines established from case S48T was performed following standard procedures as described for a case S42T [6]. In brief, genomic DNA was isolated from the primary culture as well as from cell lines and labeled with biotin-16-dUTP (Roche Applied Science, Indianapolis, IN, USA). Normal female reference DNA was isolated from peripheral lymphocytes of a healthy donor and labeled with dig-11-dUTP. After hybridization to normal metaphase spreads of a healthy donor, labeled DNA probes were detected with streptavidin-Cy2 or avidin DCS-FITC (Vector Inc., Burlingame, CA, USA) and anti-digoxigenin-Cy3/rhodamine conjugates. Slides were counterstained with 4′,6-diamidino-2-phenyl-indole (DAPI, Calbiochem, La Jolla, CA, USA) for chromosome identification. For CGH analysis, eight or more metaphases were analyzed. Averaged profiles were generated by CGH analysis software (Vysis, Downers Grove, IL, USA) from 10–15 homologous chromosomes and interpreted according to published criteria [61,62].

### 3.3. Spectral Karyotyping Analysis (SKY)

Spectral Karyotyping is a molecular cytogenetic procedure to screen the entire human genome for interchromosomal translocations by hybridization of 24 different WCP probes mixtures to metaphase spreads. We applied SKY to case S48T and identified complex aberration patterns [8]. The SKY analyses followed essentially the recommendations of the manufacturer of the reagents and the SKY imaging instrumentation (Applied Spectral Imaging (ASI), Carlsbad, CA). Briefly, fixed cells on slides were pretreated with 50 μg/mL pepsin (Amresco, Solon, OH) in 0.01N HCl for 10 min at 37 °C before immersion in phosphate buffered saline (PBS) for 5 min. The slides were then incubated in paraformaldehyde (PFA) solution (1% in PBS) for 5 min, then in PBS for 5 min. After immersion in a 70%, 80%, 100% ethanol series for 3–5 min each step, the slides were air dried. Cells on slides were denatured for 5 min at 76 °C in 70% formamide (FA)(Invitrogen, Carlsbad, CA, USA)/2 × SSC and then dehydrated in 70%, 80%, and 100% ethanol (2 min per step) before air drying.

Meanwhile, the hybridization mixture (ASI) containing 24 painting probes, each specific for one human chromosome type and labeled with combinations of five different reporter molecules was denatured for 5–6 min at 76 °C, and pre-annealed/-blocked for 30–90 min at 37 °C. The pre-blocked hybridization mixture was then applied to each slide, cover slips were place on top and sealed with rubber cement. The hybridization reaction proceeded for 18-42 h at 37 °C, before the slides were washed three times for 10 min each at 43 °C in 50% FA/2 × SSC, then twice in 2 × SSC (10 min each at 43 °C). The slides were mounted with 8 μL of 4,6-diamino-2-phenylindole (DAPI) (0.1 μg/mL) in antifade solution (0.1% p-phenylenediamine dihydrochloride (Sigma, St. Louis, MO, USA), 0.1× phosphate buffered saline (Invitrogen), 45 mM NaHCO3, 82% glycerol (Sigma), pH 8.0) and coverslipped. Metaphases images were acquired with the Spectracube system (ASI) and analyzed with SKYVIEW software [41,63].

### 3.4. Preparation of Locus-Specific DNA Probes (LSPs)

Our procedures for preparation of DNA probes from BAC/PAC clones [64,65] have been described in detail before [1,66,67]. Prior to the chromosome 9-specific FISH studies, 151 BAC clones from the Sanger Center 1 Mbp set [47] were re-arrayed on two 96-well microtiter plates (Table 3). Using information in publicly available databases (http://genome.ucsc.edu/ and http://www.ncbi.nlm.nih.gov/gquery/gquery.fcgi), we selected additional BAC clones for the long arm of chromosome 1 from the Roswell Park Cancer Institute (RPCI) library RP11 [68] and for chromosome 9. A subtelomeric clone placed in position A1 on Plate 1, GS1-41L13, is not shown in Table 3. This BAC maps to the short arm of chromosome 8 (Figure 9).

**Table 3:** BAC clones selected to map breakpoints on chromosome 9.

**Pool**	**Region**	**Clone**	**Start (bp)**	**End (bp)**	**BAC Insert Size (bp)**
9-1	9p24.3	GS1-77L23	222308	336203	113895
9-1	9p24.3	RP11-147I11	991152	1101150	109998
9-1	9p24.3	RP11-66M18	1340595	1488472	147877
9-1	9p24.3-p24.2	RP11-48M17	2136364	2296360	159996
9-1	9p24.2	RP11-320E16	2521111	2521805	694
9-1	9p24.2	RP11-509J21	3533199	3696631	163432
9-1	9p24.1	RP11-125K10	4819733	4991796	172063
9-1	9p24.1	RP11-509D8	4911574	5121406	209832
9-1	9p24.1	RP11-218I7	5993718	6146499	152781
9-1	9p24.1	RP11-106A1	6566990	6567805	815
9-1	9p24.1	RP11-283F6	7267032	7418276	151244
9-2	9p24.1	RP11-283F6	7267081	7418295	151214
9-2	9p24.1	RP11-29B9	7904520	8056890	152370
9-2	9p24.1	RP11-175E13	8398615	8557610	158995
9-2	9p23	RP11-527D15	9657611	9823754	166143
9-2	9p23	RP11-19G1	9932073	10130653	198580
9-2	9p23	RP11-23D5	11170428	11341967	171539
9-2	9p23	RP11-352F21	11389658	11588107	198449
9-2	9p23	RP11-446F13	12225005	12396287	171282
9-2	9p23	RP11-187K14	12819715	13004078	184363
9-2	9p23	RP11-413D24	13729630	13912935	183305
9-2	9p22.3	RP11-408A13	14419816	14586697	166881
9-3	9p22.3	RP11-490C5	15219945	15401987	182042
9-3	9p22.3	RP11-109M15	16141186	16325481	184295
9-3	9p22.2	RP11-132E11	16987010	17148443	161433
9-3	9p22.2	RP11-123J20	17839221	18013839	174618
9-3	9p22.1	RP11-503K16	18579957	18743091	163134
9-3	9p22.1	RP11-513M16	19310518	19506748	196230
9-3	9p21.3	RP11-15P13	20172465	20351121	178656
9-3	9p21.3	RP11-113D19	21157685	21158452	767
9-3	9p21.3	RP11-149I2	21851433	22046818	195385
9-3	9p21.3	RP11-11J1	22479595	22579721	100126
9-4	9p21.3	RP11-495L19	23376562	23557443	180881
9-4	9p21.3	RP11-33K8	24090721	24243438	152717
9-4	9p21.3	RP11-468C2	24877888	25069382	191494
9-4	9p21.2	RP11-33G16	25690187	25853227	163040
9-4	9p21.2	RP11-5P15	26681234	26681720	486
9-4	9p21.2	RP11-27J8	27417088	27590261	173173
9-4	9p21.1	RP11-20P5	28027075	28204449	177374
9-4	9p21.1	RP11-264J11	28840514	28840768	254
9-4	9p21.1	RP11-383F6	29089125	29250390	161265
9-4	9p21.1	RP11-48L13	29493271	29639053	145782
9-5	9p21.1	RP11-2G13	30199650	30364698	165048
9-5	9p13.3	RP11-573M23	34323596	34407345	83749
9-5	9p13.3	RP11-395N21	35284076	35428177	144101
9-5	9p13.3	RP11-421H8	36088495	36279930	191435
9-5	9p13.2	RP11-220I1	37065972	37242474	176502
9-5	9p13.2	RP11-113O24	38261089	38427295	166206
9-5	9p13.1	RP11-138L21	39175643	39294206	118563
9-5	9p12	RP11-38P6	42614658	42703483	88825
9-5	9p12	RP11-111G23	42933608	43076412	142804
9-6	9q13	RP11-274B18	68358409	68528389	169980
9-6	9q21.11	RP11-265B8	68778953	68779698	745
9-6	9q21.11	RP11-109D9	69487306	69676572	189266
9-6	9q21.11	RP11-141J10	70528528	70677340	148812
9-6	9q21.11	RP11-563H8	71314567	71465298	150731
9-6	9q21.12	RP11-429L21	72321408	72481088	159680
9-6	9q21.12	RP11-71A24	72848317	73017346	169029
9-6	9q21.12	RP11-401G5	73624112	73796829	172717
9-6	9q21.13	RP11-66O21	75439414	75440255	841
9-6	9q21.13	RP11-422N19	76090213	76253493	163280
9-7	9q21.13	RP11-490H9	76861448	77031282	169834
9-7	9q21.13	RP11-336N8	77969998	77970521	523
9-7	9q21.13	RP11-174K23	78534808	78716286	181478
9-7	9q21.2	RP11-362L2	79355248	79356032	784
9-7	9q21.2	RP11-280K20	80042734	80187829	145095
9-7	9q21.2	RP11-384P5	80182461	80364063	181602
9-7	9q21.2-q21.3	1 RP11-66D1	80991481	81138354	146873
9-7	9q21.31	RP11-432M2	82008792	82208346	199554
9-7	9q21.31	RP11-541F16	82662629	82822736	160107
9-7	9q21.31	RP11-439A18	83330646	83525574	194928
9-8	9q21.31	RP1-292F10	83899162	83988222	89060
9-8	9q21.32	RP11-59M22	84220295	84377175	156880
9-8	9q21.32	RP11-172F7	85287618	85288314	696
9-8	9q21.32	RP11-280P22	85960413	86094507	134094
9-8	9q21.32-q21.33	RP11-276H19	86827154	86980390	153236
9-8	9q21.33	RP11-423O13	86923188	87098245	175057
9-8	9q21.33	RP11-40C6	87248292	87415074	166782
9-8	9q21.33	RP11-249H20	87325937	87486007	160070
9-8	9q21.33	RP11-65B23	87486049	87654534	168485
9-8	9q21.33	RP11-345K9	87869665	88066130	196465
9-9	9q21.33	RP11-176L21	88644066	88799441	155375
9-9	9q21.33	RP11-8B23	89927165	89927994	829
9-9	9q21.33-q22.1	RP11-555F9	90225650	90402024	176374
9-9	9q22.1	RP11-440G5	91210454	91381302	170848
9-9	9q22.2	RP11-19J3	92321634	92489352	167718
9-9	9q22.2	RP11-30L4	93288305	93459139	170834
9-9	9q22.31	RP11-333I7	94415600	94590889	175289
9-9	9q22.31	RP11-279I21	94473904	94655715	181811
9-9	9q22.31	RP11-435O5	95213051	95402627	189576
9-9	9q22.31	RP11-160D19	95433765	95598231	164466
9-10	9q22.31	RP11-240L7	96060259	96229878	169619
9-10	9q22.32	RP11-23J9	97120587	97286003	165416
9-10	9q22.32	RP11-23B15	97623563	97784334	160771
9-10	9q22.32	RP11-92C4	98644256	98794171	149915
9-10	9q22.32	RP11-192E23	98744783	98745226	443
9-10	9q22.32	RP11-96L7	98922778	99098674	175896
9-10	9q22.32-q22.33	RP11-547C13	99270898	99449952	179054
9-10	9q22.33	RP11-463M14	99548194	99709828	161634
9-10	9q22.33	RP11-463M14	99548222	99709762	161540
9-10	9q22.33	RP11-208F1	100050139	100197864	147725
9-11	9q22.33	RP11-80H12	100788649	100957646	168997
9-11	9q22.33	RP11-75J9	101521451	101680519	159068
9-11	9q22.33	RP11-318L4	103254007	103418980	164973
9-11	9q31.1	RP11-185E13	103623573	103794095	170522
9-11	9q31.1	RP11-31J20	104568352	104754723	186371
9-11	9q31.1	RP11-287A8	105223738	105396654	172916
9-11	9q31.1	RP11-540H22	106247901	106435604	187703
9-11	9q31.1	RP11-438P9	107357324	107357981	657
9-11	9q31.2	RP11-400A24	108279653	108468171	188518
9-11	9q31.2	RP11-388N6	109146376	109360971	214595
9-12	9q31.2	RP11-470J20	109953950	110131864	177914
9-12	9q31.2	RP11-202G18	110955506	111132187	176681
9-12	9q31.3	RP11-570D4	111731038	111917067	186029
9-12	9q31.3	RP11-88M9	112540764	112727061	186297
9-12	9q31.3	RP11-534I8	113659179	113845753	186574
9-12	9q31.3	RP11-78H18	114647112	114805811	158699
9-12	9q32	RP11-279J9	114917908	115093107	175199
9-12	9q32	RP11-445L6	114960260	115162581	202321
9-12	9q32	RP11-445L6	114981548	115162653	181105
9-12	9q32	RP11-382H18	115128372	115297394	169022
9-13	9q32	RP11-404K23	115288454	115472575	184121
9-13	9q32	RP11-58C3	115951715	116121055	169340
9-13	9q32	RP11-67K19	116408159	116563591	155432
9-13	9q32	RP11-388N2	117294858	117470365	175507
9-13	9q33.1	RP11-451E16	118116160	118310814	194654
9-13	9q33.1	RP11-574M5	118953289	119134969	181680
9-13	9q33.1	RP11-28O4	119071486	119072140	654
9-13	9q33.1	RP11-360A18	119775925	119958173	182248
9-13	9q33.1	RP11-165P4	120891105	121069469	178364
9-13	9q33.1	RP11-477J21	120973024	121178266	205242
9-14	9q33.1	RP11-429D3	121691418	121864392	172974
9-14	9q33.2	RP11-137O6	122808959	122994424	185465
9-14	9q33.2	RP11-417B4	123491878	123688856	196978
9-14	9q33.2	RP11-101K10	124167663	124330481	162818
9-14	9q33.2	RP11-269P11	125271849	125447742	175893
9-14	9q33.3	RP11-205K6	126296031	126460599	164568
9-14	9q33.3	RP11-373J8	127282486	127499995	217509
9-14	9q33.3	RP11-545E17	128541257	128707904	166647
9-14	9q33.3	RP11-202H3	129858645	130045763	187118
9-15	9q34.11	RP11-57C19	130510169	130683466	173297
9-15	9q34.11	RP11-83J21	130670998	130857947	186949
9-15	9q34.11	RP11-143H20	130881497	131058128	176631
9-15	9q34.11	RP11-5N16	132007228	132007771	543
9-15	9q34.11	RP11-295G24	132650995	132860166	209171
9-15	9q34.12	RP11-153P4	133571331	133750415	179084
9-15	9q34.13	RP11-399H11	135198232	135419560	221328
9-15	9q34.2	RP11-83N9	136207935	136362829	154894
9-15	9q34.3	RP11-417A4	137679200	137871989	192789
9-15	9q34.3	GS1-135I17	138168343	138274031	105688

**Figure 11:**
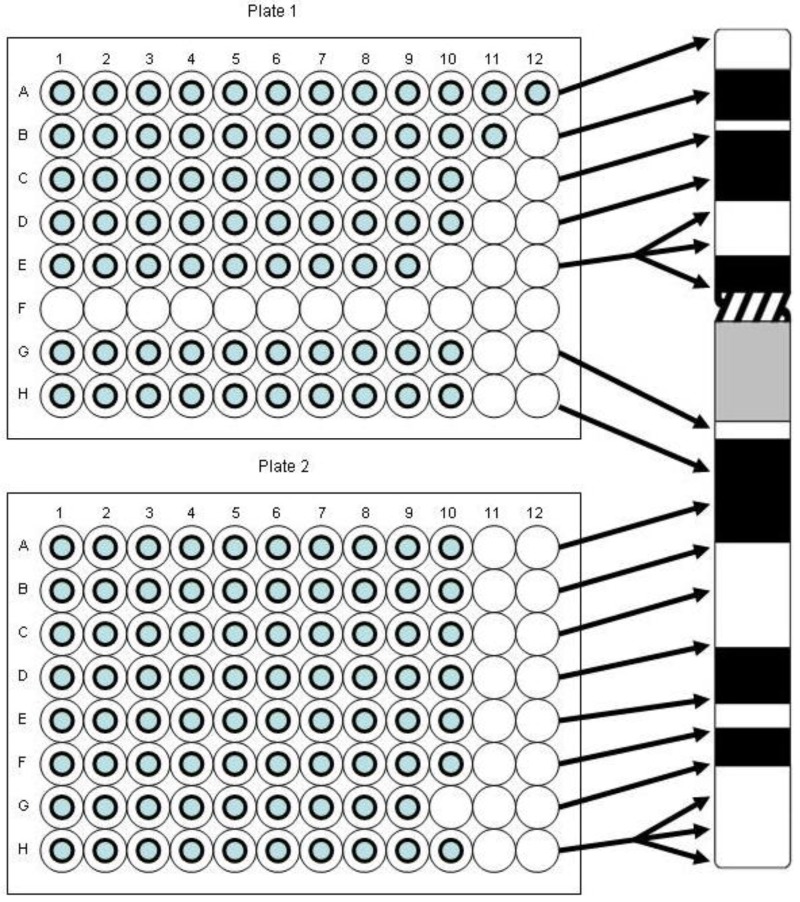
Our BAC probe pooling strategy. Please note that the BAC clone in position A1 on Plate 1 was not used in the study of thyroid tissue described here.

Individual clones were arranged so that the entire chromosome 9-specific clone set was contained on two 96-well plates in 15 rows termed “pools” with 9-12 clones per pool in individual wells (Figure 11). This created pools “9-1” to “9-15”, each of which covers a few megabase pairs (Mbp) of DNA on chromosome 9 roughly equivalent to chromosomal bands. Pools 9-1 to 9-5 (a total of 51 clones) and pools 9-6 to 9-15 (a total of 99 clones) map to the short and long arm of chromosome 9, respectively. The pool coverage ranges from 3.85 Mbp for pool 9-8 to 12.88 Mbp for pool 9-5. When large numbers of clones were grown, overnight cultures were done individually in 2 mL of Luria broth (LB) medium in 96 deep well plates (Beckman, City of Hope, CA). Fewer individual clones were grown overnight in up to 20 mL of Luria broth (LB) medium [69] containing 12.5 μg/mL chloramphenicol (Sigma) and the DNA was extracted using an alkaline lysis protocol as described [70,71]. For preparation of DNA pools or “super-pools”, *i.e.*, combination of two or more pools, clones were grown individually and pooled prior to DNA extraction. Quality control and quantification of the DNA was typically done by agarose gel electrophoresis and fluorometry, respectively.

All DNA probes were prepared by random priming (BioPrime kit, Invitrogen, Carlsbad, CA, USA) incorporating biotin-14-dCTP (part of the BioPrime kit), digoxigenin-11-dUTP (dig-11-dUTP, Roche Applied Science), fluorescein-12-dUTP (Roche Applied Science), Cy5-dUTP (Amersham, Arlington Heights, IN, USA) or Cy5.5-dCTP (Perkin Elmer, Wellesley, MA, USA) [3,72,73]. Between 0.5 μL and 3 μL of each probe along with of 4 μL human COT1™ DNA (1 mg/mL, Invitrogen) and 1 μL salmon sperm DNA (20 mg/mL, 3′-5′, Boulder, CO, USA) were precipitated with 1 μL glycogen (Roche Applied Science, 1 mg/mL) and 1/10 volume of 3 M sodium acetate in 2 volumes of 2-propanol, air dried and resuspended in 3 μL water, before 7 μL of hybridization master mix (78.6% formamide (FA), 14.3% dextran sulfate in 2.9× SSC, pH 7.0) were added. Thus, the total volume of the hybridization mixture reached 10 μL. Hybridization and detection of bound probes followed our published procedures [1–8,43]. Biotinylated and digoxigenin-labeled probes were detected with avidin-FITC (Vector, Burlingame, CA, USA; green fluorescence) and rhodamine-conjugated antibodies to digoxigenin (Roche Applied Science; red fluorescence).

In this communication, we will refer to the combination of all 150 BAC-derived DNA probes as whole chromosome painting (WCP) probe and call combinations of pools 9.1–9.5 and 9.6–9.15 “chromosome arm probes (CAP)” for chromosome 9p and 9q, respectively. To investigate chromosome 9 rearrangements in S48T with higher resolution, we labeled DNA extracted from 9p-specific clone pools and chromosome 9q-specific, adjacent pairs of pools with 5 different fluorochromes, and refer to these probes as “chromosomal rainbow probes (CRP)”.

## 4. Conclusions

In many known instances, recurrent chromosomal rearrangements are not just random events in solid tumors, but become apparent once cells carrying these abnormalities gain growth advantages over other clones. Thus, knowledge regarding the physical location of translocation breakpoints, activation of proto-oncogenes or inactivation of tumor suppressor genes may provide crucial information for a better staging of tumors and/or the definition of treatment regimens for individualized anti-tumor therapy.

Technical approaches described in this communication outline rapid and thus cost-efficient ways to analyze a patient's karyotype and reveal abnormalities within a matter of days. Utilizing resources that have been generated in the course of the International Human Genome Project, such as BAC libraries providing multi-fold coverage of the human genome, and avoiding the need for costly equipment, an average lab with basic instrumentation will now be able to perform and rapidly conclude high resolution physical mapping experiments of cancer genomes.

## Disclaimer

This document was prepared as an account of work sponsored by the United States Government. While this document is believed to contain correct information, neither the United States Government nor any agency thereof, nor The Regents of the University of California, nor any of their employees, makes any warranty, express or implied, or assumes any legal responsibility for the accuracy, completeness, or usefulness of any information, apparatus, product, or process disclosed, or represents that its use would not infringe privately owned rights. Reference herein to any specific commercial product, process, or service by its trade name, trademark, manufacturer, or otherwise, does not necessarily constitute or imply its endorsement, recommendation, or favoring by the United States Government or any agency thereof, or The Regents of the University of California. The views and opinions of authors expressed herein do not necessarily state or reflect those of the United States Government or any agency thereof, or The Regents of the University of California.
